# Distinctive behavioral and cellular responses to fluoxetine in the mouse model for Fragile X syndrome

**DOI:** 10.3389/fncel.2014.00150

**Published:** 2014-05-28

**Authors:** Marko Uutela, Jesse Lindholm, Tomi Rantamäki, Juzoh Umemori, Kerri Hunter, Vootele Võikar, Maija L. Castrén

**Affiliations:** ^1^Institute of Biomedicine/Physiology, University of HelsinkiHelsinki, Finland; ^2^Neuroscience Center, University of HelsinkiHelsinki, Finland; ^3^Department of Child Neurology, Hospital for Children and Adolescents, University Hospital of HelsinkiHelsinki, Finland

**Keywords:** behavior, autism, BDNF, neurogenesis, TrkB receptors

## Abstract

Fluoxetine is used as a therapeutic agent for autism spectrum disorder (ASD), including Fragile X syndrome (FXS). The treatment often associates with disruptive behaviors such as agitation and disinhibited behaviors in FXS. To identify mechanisms that increase the risk to poor treatment outcome, we investigated the behavioral and cellular effects of fluoxetine on adult *Fmr1* knockout (KO) mice, a mouse model for FXS. We found that fluoxetine reduced anxiety-like behavior of both wild-type and *Fmr1* KO mice seen as shortened latency to enter the center area in the open field test. In *Fmr1* KO mice, fluoxetine normalized locomotor hyperactivity but abnormally increased exploratory activity. Reduced brain-derived neurotrophic factor (BDNF) and increased TrkB receptor expression levels in the hippocampus of *Fmr1* KO mice associated with inappropriate coping responses under stressful condition and abolished antidepressant activity of fluoxetine. Fluoxetine response in the cell proliferation was also missing in the hippocampus of *Fmr1* KO mice when compared with wild-type controls. The postnatal mRNA expression of serotonin transporter (SERT) was reduced in the thalamic nuclei of *Fmr1* KO mice during the time of transient innervation of somatosensory neurons suggesting that developmental changes of SERT expression were involved in the differential cellular and behavioral responses to fluoxetine in wild-type and *Fmr1* mice. The results indicate that changes of BDNF/TrkB signaling contribute to differential behavioral responses to fluoxetine among individuals with ASD.

## INTRODUCTION

Fragile X syndrome (FXS) is a common inherited cause of intellectual disability and a well characterized form of autism spectrum disease (ASD). The behavioral phenotype of FXS includes hyperactivity, difficulties with regulation of attention, and many features that are associated with infantile autism, including motor stereotypies, poor eye contact, social avoidance, perseverative and self-injurious behavior, and delayed speech development (Hagerman et al., 2010). It has been estimated that approximately 30% of males with FXS meet the diagnostic criteria for autism (Brown et al., 1986; Hernandez et al., 2009; Hagerman et al., 2010). Perseveration in speech and behavior in FXS resemble obsessive and compulsive behavior. Obsessive thoughts and behavior are sometimes problems for FXS individuals. FXS is caused by a loss of functional FMR1 protein (FMRP), an RNA-binding protein that interacts with many pre- and postsynaptic transcripts and regulates their translation (Darnell et al., 2011). The absence of FMRP leads to aberrances in local synaptic connections, membrane excitability, and circuit activity (Bassel and Warren, 2008; Gibson et al., 2008). Alterations of neural progenitor cell proliferation and differentiation both in developing and adult brain contribute to the pathophysiology of FXS (Castrén et al., 2005; Luo et al., 2010). Several studies indicate that brain-derived neurotrophic factor (BDNF) and its tropomyosin-related kinase B (TrkB) receptors are involved in the plasticity changes in FXS (Uutela et al., 2012; Castrén and Castrén, 2014) as well as in autism (Perry et al., 2001; Miyazaki et al., 2004; Connolly et al., 2006; Correia et al., 2011; Garcia et al., 2012).

Selective serotonin reuptake inhibitors (SSRIs) such as fluoxetine are often prescribed medications for ASD (Aman et al., 2005; Oswald and Sonenklar, 2007). Although some studies suggest that fluoxetine may be beneficial for core features of ASD in adults (Williams et al., 2013) and in individual cases and subgroups of children with autism (DeLong et al., 1998, 2002; Hollander et al., 2012), a recent meta-analysis indicates that there is not enough evidence to support the use of SSRIs in autism (Williams et al., 2013). In addition, the possible side-effects of the drug treatment are a main concern in clinics. Treatment with fluoxetine has been shown to be of benefit to some FXS individuals with autism, social anxiety, or selective mutism (Hagerman et al., 1994). However, fluoxetine may not be suitable to all individuals with FXS and it can cause mood changes, restlessness, and aggression (Hagerman et al., 2009).

Fluoxetine acts primarily as an inhibitor of serotonin transporter (SERT) and blocks serotonin uptake from the synaptic cleft into presynaptic vesicles in the central nervous system (Wong et al., 1974). Fluoxetine also inhibits a number of ion channels and may suppress excitotoxicity (Kim et al., 2013b). The mechanisms of fluoxetine action involve multiple molecular pathways, including the activation of serotonergic receptors (Banasr et al., 2004), the cAMP-CREB signaling pathway (Warner-Schmidt and Duman, 2006), and signaling pathways associated with BDNF and TrkB (Duman and Monteggia, 2006; Warner-Schmidt and Duman, 2006). The clinical antidepressant effects of fluoxetine have been shown to be mediated via changes in neurogenesis and neuronal elimination (Sairanen et al., 2005; Duman and Monteggia, 2006). In the present study, we investigated behavioral and cellular effects of long-term fluoxetine treatment on adult *Fmr1* knockout (KO) mice, a mouse model for FXS, and examined the contribution of BDNF and TrkB to fluoxetine responses in FXS.

## MATERIALS AND METHODS

### ANIMALS

*Fmr1* KO mice (B6.129P2-Fmr1tm1/Cgr/J) purchased from Jackson Laboratory (Bar Harbor, ME, USA) and maintained on the C57BL/6JOlaHsd substrain in the Animal Centre of University of Helsinki were used for the behavioral studies. Male mice at the age of 3–4 months were used. Each experimental group contained four mice. Group-housed mice were maintained under 12-h light–dark cycle (lights on from 06.00 to 18.00 h) with food and water available *ad libitum*. The behavioral experiments were carried out during light phase (between 09.00 and 16.00 h). *Fmr1*-KO mice and their WT littermates used at postnatal day 7–8 (P7-8) were on inbred FVB background (Bakker et al., 1994). Animal experiments were performed in accordance with the guidelines of the National Institutes of Health Guide for the Care and Use of Laboratory Animals and European Economic Community Council Directive. All animal procedures were approved by the Experimental Animal Ethics Committee of Finland.

### FLUOXETINE ADMINISTRATION AND CELL BIRTH STUDIES

Fluoxetine was administered via drinking water (0.10 mg/ml, about 10 mg/kg/day, Orion Pharma, Finland) and control mice received water without fluoxetine. Mice received an intraperitoneal injection of bromodeoxyuridine (BrdU, Sigma-Aldrich) at a dose of 75 mg/kg four times every 2 h (300 mg/kg total) starting 24 h before sacrifice for studies investigating the proliferation and short term survival of newborn cells in the hippocampus. Hippocampi were dissected after cervical dislocation in CO_2_ anesthesia. The BrdU labeling was detected as described previously (Wu and Castrén, 2009). Briefly, deoxyribonucleic acid was extracted from the hippocampi, denatured, and dot-blotted onto membrane. The BrdU incorporation was detected by immunostaining with mouse BrdU-specific monoclonal primary antibody (Roche, 1-299-964).

### BEHAVIORAL TESTING

Behavioral testing was performed between 9:00 AM and 4:00 PM by experimenters who were blinded to the genotypes at the time of testing.

#### Open field test

The mice were released in the corner of novel open field arena (30 × 30 cm, Med Associates, St. Albans, VT, USA) surrounded by frames with infra-red light barriers for detection of animal’s position. Horizontal and vertical activity was recorded for 30 min (light intensity ~150 lx). Peripheral zone was defined as a 6 cm-wide corridor along the wall.

#### Forced swim test

Mice were placed in a clear, 21°C water-filled cylinder (diameter, 20 cm; depth, 13 cm) for 6 min and the immobility time of the mice was measured between 2 and 6 min.

### BDNF ELISA

For the BDNF expression studies, hippocampi were collected from mice sacrificed by cervical dislocation followed by anesthesia with CO_2_. Samples were frozen on dry ice, and stored at –70°C until use. The BDNF expression was determined using BDNF ELISA (Quantikine human BDNF kit, R&D Systems) as described previously (Louhivuori et al., 2011).

### WESTERN ANALYSIS

The samples were homogenized and processed in a lysis buffer for Western analysis as previously described (Castrén et al., 2002). The protein concentration of the supernatant samples was determined using Biorad DC protein assay. The protein extracts (60 μg) were electrophoresed on 7.5% sodium dodecyl sulfate polyacrylamide minigels and transferred to 0.2 mm nitrocellulose membranes (Schleicher & Schuell) for 1 h at 400 mA. The membranes were washed 10 min in TBS, pH 7.4 (0.1 M Tris, 0.15 M NaCl) and blocked in 5% non-fat dry milk, in TBS with 0.1% Tween 20 (TBST) for 1.5 h. The incubation with rabbit anti-TrkB (1:1000, sc-11, Santa Cruz Biotechnology) at +4°C overnight was followed by washes in TBST and incubation with horseradish-peroxidase-conjugated secondary antibody (1:10000, Bio-Rad Laboratories) for 1.5 h at room temperature. Detection was performed using the enhanced chemiluminescence kit (ECL^+^^+^ kit, Amersham Biosciences) and Fuji LAS-3000 camera (Tamro Medlabs, Vantaa, Finland). Data were analyzed using NIH Image J software.

### IN SITU HYBRIDIZATION

Mouse brains at P7-8 were fixed in 4% paraformaldehyde (PFA) in phosphate buffered saline (PBS) overnight and processed for frozen sectioning. Brains were washed twice in PBS after fixation and then soaked in cryoprotective solution (30% sucrose in PBS). Brains were mounted in Tissue-Tek®(Sakura Finetek, Zoeterwoude, Netherlands), frozen on dry ice, and stored at –80°C until cut. Brains were cut in 12 μm thick sections and collected onto Superfrost® Plus microscope slides (Menzel GmbH & Co. KG, Braunschweig, Germany) with MICROM HM 550 cryostat (MICROM International GmbH, Walldorf, Germany) and the slides were stored at –80°C until use.

*In situ* hybridization with the oligonucleotide probes was performed as described by Wisden and Morris (1994). Oligonucleotides complementary to mouse SERT (5′-ATG AGG TAG TAG AGC GCC CAG GCT ATG ATG GTG TT-3′) were 3′ labeled with [α^33^P]-dATP (3000/mmol; Amerham Biosciences) to a specific activity of 6–7 × 10^-^^7^cpm/pmol using terminal deoxynucleotidyl transferase (Finnzymes). Hybridization was performed overnight (42°C) on postfixed sections in the presence of 1 × 10^6^ cpm/ml labeled probe in buffer containing 50% formamide, 4× standard saline citrate (SSC; 1×SSC: 150 mM NaCl, 15 mM sodium citrate), 10% dextran sulfate and 10 mM dithiothreitol. After overnight hybridization at 42°C, the sections were dipped into 1×SSC and then sequentially for 3 min each at room temperature in 1×SSC, 0.1×SSC, 70% ethanol, and 94% ethanol. Microscope slides were exposed to film (Fuji medical X-ray film super RX) for three weeks. ^14^C standard scale was included to every film. Hybridization signal intensities were quantified from films scanned with Fujifilm FLA-5100 scanning device.

### NEURAL PROGENITOR CULTURES

Neural progenitors were propagated from the wall of lateral ventricles of wild-type and *Fmr1* KO pups as previously described (Castrén et al., 2005). Cells were grown as free-floating aggregates referred to as neurospheres in Dulbecco’s modified Eagle’s medium F-12 nutrient mixture (DMEM/F-12) media containing B27 supplement (both from Gibco, Life Technologies Ltd.), L-glutamine (2 mM), 4-(2-hydroxyethyl)-1-piperazineethanesulfonic acid (HEPES, 15 mM), penicillin (100 U/ml), and streptomycin (100 U/ml) (all from Sigma-Aldrich), in the presence of basic fibroblastic growth factor (10 ng/ml) and epidermal growth factor (20 ng/ml) (both from PeproTech) in a 5% CO_2_-humidified incubator at +37^o^C. The culture medium was refreshed and growth factors were added three times per week. The cells were passaged by manual trituration at approximately two weeks intervals. Neuronal progenitor cells from WT and *Fmr1* KO mice were plated at a concentration of 100000 cells/10 ml plate and grown as neurospheres for 5 days. Medium was changed and growth factors last added 5 h prior to the start of treatments. Cells were treated with 1 μM fluoxetine in parallel with corresponding non-treated controls for 48 h. The cells were then collected as cell pellets and stored at –70°C until further use.

### RNA EXTRACTION AND REAL-TIME QUANTITATIVE PCR

Total RNA was extracted from frozen cells by using QIAzol (Qiagen, Valencia, CA, USA) and treated with DNaseI (Thermo Fisher Scientific Inc., Rockford, IL, USA) according to the manufacturer’s instruction. We used 2–4 μg of total RNA to synthesize cDNA using the Maxima First Strand cDNA Synthesis Kit (Thermo Fisher Scientific Inc., Rockford, IL, USA). Real-time quantitative PCR was performed using the Maxima SYBR Green qPCR Master Mix (Thermo Fisher Scientific Inc., Rockford, IL, USA) and the CFX96 Touch^TM^ detection system (Bio-Rad, Hercules, CA, USA). The primers described previously (Karpova et al., 2009) were used to amplify specific cDNA regions of transcripts: the coding region in the exon IX of the *Bdnf* gene for the total *Bdnf* mRNA (5′-GAAGGCTGCAGGGGCATAGACAAA-3′ and 5′-TACACAGGAAGTGTCTATCCTTATG-3′); the exon IV (5′-ACCGAAGTATGAAATAACCATAGTAAG-3′) and (5′-TGTTTACTTTGACAAGTAGTGACTGAA-3′), *Gapdh* (5′-GGTGAAGGTCGGTGTGAACGG-3′ and 5′-ATGTAGTTGAGGTCAATGAAGGG-3′) as a housekeeping control gene. Ct and quantitative values were calculated from each sample using CFX Manager^TM^ software (Bio-Rad, Hercules, CA, USA) and the quantitative values were normalized to the control *Gapdh* levels.

### DATA ANALYSIS

Data obtained from behavioral tests were analyzed with Statview software (SAS, Cary, NC, USA), unless specified otherwise, a two-way repeated-measures analysis of variance (ANOVA) followed by Fishers’s protected least significance *post hoc* test.

Immunoblot bands were quantified using NIH ImageJ software. All the data are presented as means ±SEM. Statistical analyses were performed using GraphPad Prism 4.0 for Windows (GraphPad Software, San Diego, CA, USA). Quantitative analysis of signals on X-ray films was performed with AIDA Image Analyzer (version 3.44.035, Raytest Isotopenmessgeräte GmbH, Straubenhardt, Germany) software. Brightness and contrast were optimized before measuring.

For comparison between two groups, Student’s *t*-test was used. Two-way ANOVA was used to reveal main effect and interaction between the factors followed by Bonferroni post hoc test. The criterion for significance was set to *P* < 0.05.

## RESULTS

### BEHAVIORAL RESPONSES OF *Fmr1* KO MICE TO FLUOXETINE IN THE OPEN FIELD TEST

**FIGURE 1:**
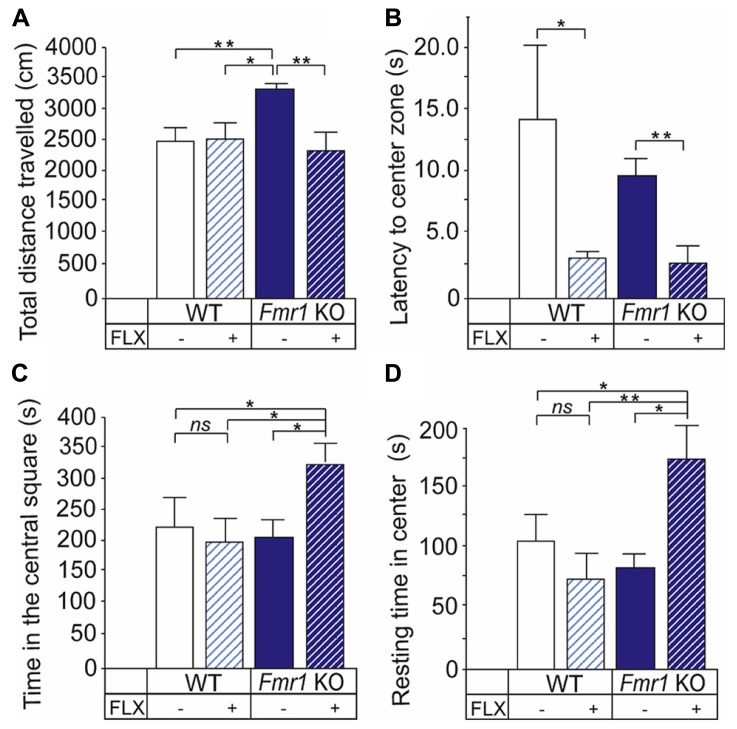
**Altered behavioral responses to fluoxetine of *Fmr1* KO mice in the open field test. (A)** *Fmr1* KO mice (*Fmr1* KO) show locomotor hyperactivity seen as longer distance traveled by the mice when compared with wild-type controls (WT). The locomotor activity of *Fmr1* KO mice was decreased to the level of WT mice Fluoxetine reduced the latency to enter the center area of the open field in both WT and *Fmr1* KO mice. **(C)** Fluoxetine increased the time that *Fmr1* KO mice spent in the central square but did not have any effects on this behavior in WT mice. **(D)** The resting time in center was increased by fluoxetine in *Fmr1* KO mice. Error bars indicate means ± SEM. *P < 0.05, **P < 0.01.

We observed a significant *Fmr1* KO genotype and fluoxetine treatment interaction (two-way ANOVA: *F*_(1,33)_ = 2,294; *P* < 0.05) in the locomotor activity in the open field test. *Fmr1* KO mice were hyperactive when compared with non-treated mice (*P* < 0.05) and treatment with fluoxetine reduced the motor activity of *Fmr1* KO mice to wild-type levels (*P* = 0.295; **Figure 1A**). There was a main effect of fluoxetine treatment (two-way ANOVA: *F*_(1,12)_ = 6.948; *P* < 0.05) but no effects of the mouse genotype (two-way ANOVA: *F*_(1,12)_ = 0.695; *P* > 0.05) on the latency to enter the center arena of the open field. Fluoxetine reduced significantly the latency in both wild-type (*P* < 0.05) and *Fmr1* KO mice (*P* < 0.05; **Figure 1B**). In addition, a significant genotype × treatment effect in the exploratory activity and unconditioned anxiety-related behavior (two-way ANOVA: *F*_(1,12)_ = 5.863; 0.05) was found. Fluoxetine increased the time that *Fmr1* KO mice spent in the central square (*P* < 0.05; **Figure 1C**) and appropriately decreased the time that the transgenic mice stayed along the perimeter (*P* < 0.05*;* data not shown) without having any effects on this behavior in wild-type mice. As shown in **Figure 1D**, particularly the resting time in center was increased (*P* < 0.05) in *Fmr1* KO mice by fluoxetine treatment. No genotype or fluoxetine effects were found on the total resting time (two-way ANOVA: *F*_(1,12)_ = 0.875; *P* > 0.05 and *F*_(1,12)_ = 2.353; *P* > 0.05, respectively).

### BEHAVIORAL RESPONSES OF *Fmr1* KO MICE TO FLUOXETINE IN THE FORCED SWIM TEST

**FIGURE 2:**
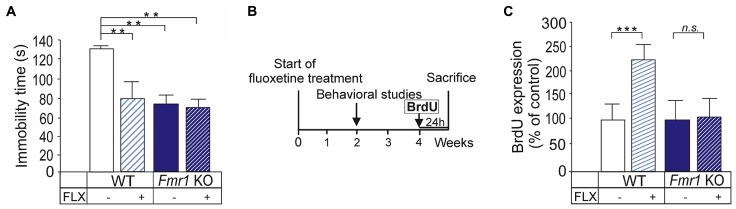
**Altered fluoxetine responses on mobility in the forced swim test and proliferation of hippocampal cells. (A)** *Fmr1* KO mice showed abnormal coping responses under stressful condition in the forced swim test and the immobility time of *Fmr1* KO mice was reduced when compared with wild-type controls. Fluoxetine reduced immobility of wild-type mice but not that of *Fmr1* KO mice. **(B)** The effects of long-term fluoxetine treatment were examined on hippocampal cell proliferation analyzed by incorporation of BrdU in newborn cells. **(C)** Fluoxetine increased significantly hippocampal cell proliferation in wild type but not in *Fmr1* KO mice after fluoxetine treatment. Error bars indicate means ± SEM. **P < 0.01, ***P < 0.001.

We investigated the antidepressant effects of fluoxetine on the phenotype of *Fmr1* KO mice by submitting the mice to the forced swim test, which estimates behavioral despair under stressful and inescapable conditions, and it is widely used screening test of antidepressant drugs to assess their antidepressant activity (Porsolt et al., 1977; Cryan et al., 2002; Prut and Belzung, 2003). In this test, wild-type mice respond to antidepressants by reducing their immobility time (Porsolt et al., 1977). We found a significant *Fmr1* KO genotype and long-term fluoxetine treatment interaction (two-way ANOVA: *F*_(1,12)_ = 11,211; *P* < 0.01). The swimming immobility of *Fmr1* KO mice was decreased (*P* < 0.001) when compared with WT littermates without any treatment (**Figure 2A**). Fluoxetine administration reduced (*P* < 0.01) immobility time of wild-type mice but had no effect on this immobility score of *Fmr1* KO mice (**Figure 2A**).

### EFFECTS OF FLUOXETINE ON CELL PROLIFERATION IN THE HIPPOCAMPUS OF *Fmr1* KO MICE

The stimulatory effect of fluoxetine on progenitor cell proliferation is implicated to its therapeutic effects. We assessed the short-term effect of fluoxetine on cell proliferation by the BrdU staining in the hippocampus 24 h after intraperitoneal BrdU injections (**Figure 2B**). As shown in **Figure 2C**, treatment with fluoxetine increased the BrdU staining 2.2-fold in wild-type mice but did not have any effects on the BrdU expression in the hippocampus of *Fmr1* KO mice (ANOVA: *F*_(3,14)_ = 5,117; *P* < 0.05). The data indicate that the normal response to fluoxetine on proliferation rate was missing in the absence of FMRP.

### RESPONSES TO FLUOXETINE IN THE EXPRESSION OF BDNF AND TrkB IN THE ABSENCE OF FMRP

**FIGURE 3:**
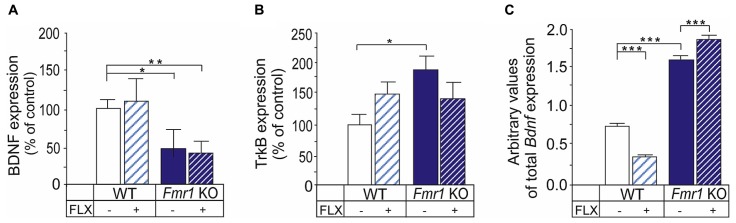
**Effects of fluoxetine on the BDNF and TrkB protein expression in the hippocampus of *Fmr1* KO mice. (A)** The BDNF protein expression was reduced in the hippocampus of *Fmr1* KO mice (*Fmr1* KO) when compared to that of wild-type mice (WT). The expression levels were not changed significantly after treatment with fluoxetine under our experimental conditions. **(B)** The TrkB receptor protein was significantly increased in the hippocampus of *Fmr1* KO mice when compared to WT controls. Fluoxetine did not have any significant effects on the TrkB expression in WT or *Fmr1* KO mice. **(C)** The expression of *Bdnf* mRNA was increased in undifferentiated neural progenitors derived from *Fmr1* KO mice when compared with WT controls and the responses to fluoxetine treatment were different in WT and transgenic progenitors. Error bars indicate means ± SEM. *P < 0.05, **P < 0.01, ***P < 0.001.

BDNF/TrkB signaling is implicated in the fluoxetine effects and chronic, but not acute, fluoxetine treatment increase BDNF in the rodent brain (Nibuya et al., 1995; Duman and Monteggia, 2006). The expression of BDNF was reduced in the hippocampus of *Fmr1* KO mice when compared with wild-type controls (**Figure 3A**) as shown previously in older *Fmr1* KO mice (Uutela et al., 2012). Treatment with fluoxetine did not have any significant effects on the BDNF protein expression in the hippocampus of wild-type or *Fmr1* KO mice in our experimental setting (**Figure 3A**). The expression of TrkB receptors was increased in the hippocampus of the *Fmr1* KO mice when compared with wild-type controls (*P* < 0.05), suggesting a role for TrkB in altered fluoxetine responses in FXS (**Figure 3B**). There was a tendency toward increased TrkB protein in the wild-type hippocampus after fluoxetine treatment and the expression of TrkB protein remained higher in the hippocampus of *Fmr1* KO than in wild-type controls after treatment but the effects of fluoxetine on the TrkB protein expression did not reach the level of significance (**Figure 3B**).

Our previous studies have shown that the dendritic targeting and expression of *Bdnf* mRNA are increased in cortical and hippocampal neurons of *Fmr1* KO (Louhivuori et al., 2011). We examined responses to fluoxetine on *Bdnf* mRNA levels in undifferentiated cortical progenitors derived from *Fmr1* KO mice. We found that the basal expression level of the total *Bdnf* mRNA in progenitors lacking FMRP was significantly higher than that in wild-type progenitors (genotype, *F*_(1,20)_ = 1148.5; *P* < 2.0e–16; **Figure 3C**). A two-way ANOVA showed that there was an interaction between genotype and drug treatment (genotype × treatment interaction, *F*_(1,20)_ = 100.0, *P* = 3.16e–09), and fluoxetine treatment reduced total *Bdnf* mRNA in wild-type progenitor cultures whereas the expression was increased by fluoxetine in cultures derived from *Fmr1* KO mice. The expression of exon IV transcripts correlated with that of total *Bdnf* mRNA (data not shown), but its large variation due to a low expression level suggested that promoter IV-driven *Bdnf* transcription was not utilized significantly in proliferating undifferentiated neural progenitors.

### SERT EXPRESSION IN POSTNATAL BRAIN OF *Fmr1* KO MOUSE

**FIGURE 4:**
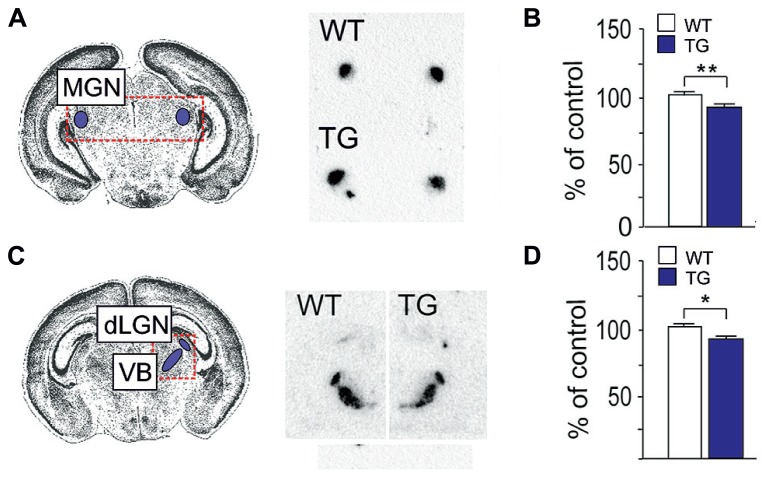
**Expression of SERT mRNA in the thalamic nuclei of *Fmr1* KO mice at P7-8. (A)** The location of the medial geniculate nucleus (MGN) in the Nissl-stained brain section and radioactive *in situ* hybridization with SERT showing the nucleus in the brain sections of a wild-type (WT) and *Fmr1* KO transgenic (TG) mouse. **(B)** A bar graph representing the decrease in the signal intensity of the SERT expression in the MGN of TG mice when compared with that of WT mice. **(C)** The location of the dorsal lateral geniculate nucleus (dLGN) and ventrobasal nucleus (VB) in the Nissl-stained brain section and the *in situ* SERT hybridization signal in the nuclei in the brain sections of a WT and TG mouse. **(D)** A bar graph representing the ratio of the signal intensities of the SERT expression in the dLGN to that in the VB of WT and TG mice. *n*(WT) = 7, *n*(TG) = 7. Error bars indicate means ± SEM. *P < 0.05, **P < 0.01.

The early development of serotonergic system has important functions in cortical maturation and plasticity (Vitalis and Parnavelas, 2003). Changes in the SERT expression during early brain development induce long-lasting behavioral alterations that associate with changes of responses to fluoxetine and expression of BDNF and TrkB (Karpova et al., 2009; Kiryanova et al., 2013). Transient SERT expression mediates innervation and the uptake of serotonin by axons and terminals of thalamic sensory neurons at P1–P10 before the total maturation of serotonergic system (Lebrand et al., 1996). We examined the SERT mRNA expression in the sensory relay nuclei of the thalamus of *Fmr1* KO mice at P7-8. The SERT expression was slightly but significantly reduced (90% of control, *P* < 0.003) in the medial geniculate nucleus (MGN) of the auditory relay in *Fmr1* KO mice when compared with wild-type controls (**Figures 4A,B**). Signal intensities in the dorsal lateral geniculate nucleus (dLGN) of the visual in *Fmr1*-KO mouse relay showed a tendency to decreased levels and the ratio of the SERT mRNA expression in dLGN to that in the ventrobasal nucleus (VB) of the somatosensory relay (90% of control, *P* < 0.048) was significantly reduced when compared with wild-type controls (**Figures 4C,D**) suggesting dysregulation of serotonin-dependent developmental processes in FXS.

## DISCUSSION

### *Fmr1* KO MICE AS A MODEL FOR AUTISM FOR FLUOXETINE STUDIES

ASD consists of a range of complex neurodevelopmental disorders, characterized by aberrant reciprocal social interactions, impaired communication, and stereotyped repetitive behaviors with narrow restricted interests. ASD varies in character and severity. The clinical phenotypes reflect heterogeneity of genetic/epigenetic/environmental factors which may contribute to alterations in developmental processes and neuronal plasticity that associate with defects in synapse and neuronal network function in autism (Hughes, 2009). A genetic association among autism and the *TrkB* gene (Correia et al., 2011), abnormal blood BDNF levels in children with autism (Nelson et al., 2001; Miyazaki et al., 2004; Connolly et al., 2006; Iughetti et al., 2011), and increased BDNF protein expression in postmortem brain tissue of autistic individuals (Perry et al., 2001; Garcia et al., 2012) suggests that BDNF/TrkB signaling plays a role in the pathophysiology of autism. FXS is the cause of autism in 2–6% of all children diagnosed with autism and the syndrome is the best characterized form of ASD (Dölen and Bear, 2009). FXS is caused by a loss of functional FMRP and *Fmr1* KO mice recapitulate the main features of the human FXS (Hagerman et al., 1994). Studies of *Fmr1* KO mice have revealed that BDNF/TrkB signaling is involved in the alterations of neurogenesis and synapse function in FXS (Louhivuori et al., 2011; Uutela et al., 2012). Here, we show aberrant behavioral and cellular responses to fluoxetine in *Fmr1* KO mice. We show that the aberrant responses associate with alterations in the expression of BDNF and TrkB receptors. Furthermore, a reduced transient SERT mRNA expression in the thalamic nuclei of *Fmr1* KO mice suggests developmental changes in the maturation of the serotonin system that can have long-lasting effects on the behavior.

### ALTERATIONS OF BEHAVIORAL EFFECTS OF FLUOXETINE IN FXS MICE

We observed that fluoxetine reduced the latency of *Fmr1* KO and wild-type mice to enter the center area in the open field test indicating reduced anxiety in both mouse groups. Fluoxetine normalized the locomotor hyperactivity characteristics of *Fmr1* KO mice (Bakker et al., 1994; Peier et al., 2000; Spencer et al., 2005; Mineur et al., 2006) and increased the exploratory activity of these mice, seen as longer time that the mice stayed in the center of the open field when compared to that of fluoxetine-treated wild-type mice. Fluoxetine did not display this type of anxiolytic effect in wild-type mice. The behavioral response to fluoxetine in *Fmr1* KO mice may correlate with disinhibited behaviors and agitation which are known side-effects of fluoxetine treatment in FXS individuals. In the forced swim test, *Fmr1* KO mice showed reduced behavioral despair under the stressful condition when compared with wild-type mice. Fluoxetine had no effects on the immobility time of *Fmr1* KO mice suggesting that the normal antidepressant effect of fluoxetine was missing in the absence of FMRP.

### THE ABSENCE OF FMRP AFFECTS CELLULAR RESPONSES TO FLUOXETINE

We found that the aberrant behavioral responses to fluoxetine in *Fmr1* KO mice correlated with alterations of cellular responses. Fluoxetine did not increase the proliferation of hippocampal cells in *Fmr1* KO mice like is normally seen in wild-type mice. The responses in cell proliferation and neurogenesis are implicated particularly in the antidepressant effects of fluoxetine. The forced swim test is used to assess the antidepressant activity of drugs, and defects in cell proliferation responses in *Fmr1* KO mice are consistent with the unresponsiveness to fluoxetine in the forced swim test. BDNF/TrkB signaling plays an essential role for the antidepressant effects of fluoxetine (Saarelainen et al., 2003; Monteggia et al., 2007; Ibarguen-Vargas et al., 2009). Behavioral effects of fluoxetine are blunted in animals with reduced BDNF expression in the central nervous system (Saarelainen et al., 2003; Ibarguen-Vargas et al., 2009). The expression of BDNF shows age-dependent changes in murine brain and temporal alterations of BDNF expression have been found in *Fmr1* KO mouse brain. The reduced expression of hippocampal BDNF protein in the *Fmr1* KO male mice at the age of 3–4 months in the present study is in agreement with an enhanced age-dependent decay of BDNF expression in the absence of FMRP (Uutela et al., 2012) and unresponsiveness to fluoxetine in the forced swim test. However, previous studies have revealed that the expression of BDNF protein is increased in the hippocampus of young *Fmr1* KO mice (Louhivuori et al., 2011; Uutela et al., 2012). We found previously an increased expression and dendritic targeting of *Bdnf* mRNAs in neurons of *Fmr1* KO mice (Louhivuori et al., 2011). Here, we showed that the *Bdnf* mRNA expression is increased in FMRP-deficient neural progenitors which express normal levels of BDNF protein (Louhivuori et al., 2011) and that the fluoxetine responses are also affected on mRNA levels in these cells.

The dynamic alterations of BDNF expression levels in *Fmr1* KO mice contribute to a behavioral phenotype that differs from the phenotype of *Bdnf* ^+^^/-^ mice with reduced BDNF expression (Uutela et al., 2012). *Bdnf* ^+^^/^^-^ mice are indistinguishable from wild-type mice in behavioral tests investigating anxiety, fear-associated learning, behavioral despair, and spatial learning (Kernie et al., 2000; MacQueen et al., 2001). In early adulthood, *Bdnf* ^+^^/^^-^ mice show aggressiveness that has been linked with dysfunction of serotonergic neurons (Lyons et al., 1999). Defects in associative learning and reduced startle responses at higher intensities are consistent findings in *Fmr1* KO mice but not seen in *Bdnf* ^+^^/^^-^ mice (Uutela et al., 2012), whereas locomotor hyperactivity is characteristics of *Fmr1* KO mice that may be seen in *Bdnf* ^+^^/^^-^ mice when stressed (Kernie et al., 2000; Rios et al., 2001). Reduced non-social but increased social anxiety have been reported in *Fmr1* KO mice (Bakker et al., 1994; Peier et al., 2000; Mineur et al., 2002; Spencer et al., 2005; Mineur et al., 2006) but the anxiety phenotype of *Fmr1* KO mice has not been consistent in all studies (Van Dam et al., 2000; Mineur et al., 2002; Nielsen et al., 2002; Zhao et al., 2005; Bernardet and Crusio, 2006).

Reduced behavioral despair in adult *Fmr1* KO mice in the forced swim test was associated with increased hippocampal TrkB receptors. Similarly, mice with overexpression of TrkB in neurons show reduced behavioral despair (Koponen et al., 2005). BDNF and TrkB are implicated in learning and memory processes, including acquisition of fear learning within amygdala (Rattiner et al., 2004). Overexpression of TrkB in transgenic mice reduces anxiety and the increased TrkB expression in *Fmr1* KO mice was likely linked with the reduced anxiety. Neuronal release of BDNF can alter anxiety-like behaviors in mice (Berton et al., 2006; Chen et al., 2006) and age-dependent changes in the expression of BDNF observed in the brain of *Fmr1* KO mice (Uutela et al., 2012) could at least partially explain the alterations seen in the anxiety phenotype in different studies. Fluoxetine displayed an abnormal anxiolytic effect that did not associate with any significant changes in the TrkB expression in the hippocampus of *Fmr1* KO mice in our experimental setting and further studies are needed to explore the regulation and functional responses of TrkB receptors after treatment with fluoxetine in different experimental conditions in FXS mice.

There is evidence that BDNF signaling is critical for the normal development and function of central serotonergic neurons (Lyons et al., 1999). Reduced levels of endogenous BDNF cause alterations of serotonergic receptor expression but brain serotonin levels and fiber density are normal in *Bdnf* ^+^^/^^-^ mice at early age. We found that the postnatal SERT mRNA expression was reduced in the thalamic nuclei of *Fmr1* KO mice during the time of transient innervation of somatosensory neurons indicating developmental changes in the serotonergic system that contribute to alterations of BDNF/TrkB signaling and behavioral responses in adult *Fmr1* KO mice. Previously, defects of AMPA receptor GluR1 subtype surface insertion have been shown after inhibition of 5-HT2A receptor also indicating defects in serotonergic system in FXS (Xu et al., 2012). Changes in the SERT mRNA expression in the sensory thalamic nuclei during postnatal period are consistent with alterations of developmental plasticity in both visual and auditory systems of *Fmr1* KO mice (Dolen et al., 2007; Kim et al., 2013a). Temporal and spatial changes of serotonin expression during early development may cause long-lasting behavioral alterations in FXS. Indeed, targeting SERT expression by fluoxetine during postnatal development results in reduced behavioral despair in adult mice as seen in *Fmr1* KO mice (Karpova et al., 2009).

### FLUOXETINE TREATMENT IN ASD

Fluoxetine is often used to treat individuals with ASD (Aman et al., 2005; Oswald and Sonenklar, 2007) and its effects have been evaluated in several clinical studies. A recently published meta-analysis does not support the use of SSRIs in autism (Williams et al., 2013). However, positive effects of fluoxetine on core autistic symptoms have been shown in individual cases and subgroups of autistic children and in adults with ASD (DeLong et al., 1998, 2002; Makkonen et al., 2011; Hollander et al., 2012). Anxiety and obsessive–compulsive symptoms which associate with autism can be ameliorated by fluoxetine in adult ASD (Buchsbaum et al., 2001; Hollander et al., 2012). In children, beneficial effects have been particularly shown in language impairment. Relatively few side-effects were observed over a 12-week fluoxetine treatment period (Hollander et al., 2012) but long-time consequences of fluoxetine treatment on human brain maturation are not known.

Improved understanding of distinct molecular mechanisms linked to SSRI action in ASD could facilitate optimal pharmacological intervention of individuals with ASD. Dysregulated serotonergic signaling in autism is supported by platelet hyperserotonemia in some of ASD individuals (Piven et al., 1991). Furthermore, linkage studies have identified ASD candidate genes in serotonergic pathways, including the gene that encodes SERT (SLC6A4) (Devlin et al., 2005; Brune et al., 2006). Chronic treatment with fluoxetine enhances serotonergic transmission that may activate mechanisms involved in regulation of intracortical inhibitory–excitatory balance and reduced γ -aminobutyric acid (GABA) signaling is reported by fluoxetine treatment (Maya-Vetencourt et al., 2008).

In the present study, we examined effects of long-term fluoxetine treatment in FXS that represents a monogenic cause of ASD. We observed aberrances of behavioral fluoxetine responses which correlated with alterations of BDNF and TrkB expression. Alterations of both excitatory and inhibitory neurotransmission are implicated in FXS and the outcome of fluoxetine treatment on function of neuronal circuits in FXS is difficult to predict. Enhanced explorative activity of *Fmr1* KO mice after fluoxetine treatment is in agreement with activation seen as restlessness, mood changes, and disinhibited behaviors in about 20% of individuals with FXS (Hagerman et al., 1994). The present study suggests that molecular mechanisms underlying ASD may associate with developmental changes that influence fluoxetine responses. Further studies are needed to investigate genetic and epigenetic factors which modulate responses to fluoxetine in ASD more in detail.

## Conflict of Interest Statement

The authors declare that the research was conducted in the absence of any commercial or financial relationships that could be construed as a potential conflict of interest.
